# Prospects and Advances in Adoptive Natural Killer Cell Therapy for Unmet Therapeutic Needs in Pediatric Bone Sarcomas

**DOI:** 10.3390/ijms24098324

**Published:** 2023-05-05

**Authors:** Halin Bareke, Adrián Ibáñez-Navarro, Pilar Guerra-García, Carlos González Pérez, Pedro Rubio-Aparicio, Diego Plaza López de Sabando, Ana Sastre-Urgelles, Eduardo José Ortiz-Cruz, Antonio Pérez-Martínez

**Affiliations:** 1 Translational Research Group in Pediatric Oncology, Haematopoietic Transplantation and Cell Therapy, Hospital La Paz Institute for Health Research, IdiPAZ, La Paz University Hospital, 28046 Madrid, Spain; halin.bareke@gmail.com (H.B.); adrianibnavarro@gmail.com (A.I.-N.); 2 Department of Pediatric Hemato-Oncology, La Paz University Hospital, 28046 Madrid, Spain; 3 Department of Orthopedic Surgery and Traumatology, La Paz University Hospital, 28046 Madrid, Spain; 4 School of Medicine, Autonomous University of Madrid, 28046 Madrid, Spain

**Keywords:** pediatric bone sarcoma, osteosarcoma, Ewing sarcoma, NK cells, adoptive cell therapy, immunotherapy, tumor microenvironment, immune evasion

## Abstract

Malignant bone tumors are aggressive tumors, with a high tendency to metastasize, that are observed most frequently in adolescents during rapid growth spurts. Pediatric patients with malignant bone sarcomas, Ewing sarcoma and osteosarcoma, who present with progressive disease have dire survival rates despite aggressive therapy. These therapies can have long-term effects on bone growth, such as decreased bone mineral density and reduced longitudinal growth. New therapeutic approaches are therefore urgently needed for targeting pediatric malignant bone tumors. Harnessing the power of the immune system against cancer has improved the survival rates dramatically in certain cancer types. Natural killer (NK) cells are a heterogeneous group of innate effector cells that possess numerous antitumor effects, such as cytolysis and cytokine production. Pediatric sarcoma cells have been shown to be especially susceptible to NK-cell-mediated killing. NK-cell adoptive therapy confers numerous advantages over T-cell adoptive therapy, including a good safety profile and a lack of major histocompatibility complex restriction. NK-cell immunotherapy has the potential to be a new therapy for pediatric malignant bone tumors. In this manuscript, we review the general characteristics of osteosarcoma and Ewing sarcoma, discuss the long-term effects of sarcoma treatment on bones, and the barriers to effective immunotherapy in bone sarcomas. We then present the laboratory and clinical studies on NK-cell immunotherapy for pediatric malignant bone tumors. We discuss the various donor sources and NK-cell types, the engineering of NK cells and combinatorial treatment approaches that are being studied to overcome the current challenges in adoptive NK-cell therapy, while suggesting approaches for future studies on NK-cell immunotherapy in pediatric bone tumors.

## 1. Introduction

Pediatric cancers make up a small percentage of all the cancer cases worldwide, but they are still a leading cause of non-communicable disease-associated mortality in children [1]. These cancers have both some unique challenges and some challenges that they share with adult cancers. As with adult cancers, relapsed and refractory tumors generally have a poor prognosis with limited treatment options. Similar to adult cancers, pediatric cancers also display heterogeneity within a cancer type, however, the low number of pediatric patients makes it particularly difficult to characterize the subtypes [2]. The number of new drugs approved for pediatric cancers is relatively low due to numerous factors, including the unique considerations for this population for clinical trials and the low patient numbers [3]. The extrapolation of the results from clinical trials on adults is tricky because the safety and efficiency profiles of drugs can show differences between these two groups. Furthermore, the long-term sequelae of the treatments, including the increased risk of secondary malignancy and long-term disability, are especially important in the pediatric population, given that these patients are in the initial stages of their lives. Therefore, there is a need for developing novel and accessible treatments for pediatric cancers by designing preclinical and clinical studies that are tailored to the needs of these cancers.

Malignant bone tumors are aggressive tumors with a high tendency to metastasize. The most common types of malignant bone tumors are Ewing sarcoma (EWS), osteosarcoma (OS), and chondrosarcoma [4]. Apart from chondrosarcoma, these cancers are most frequently observed in the pediatric population [4]. Nearly half of pediatric patients with malignant bone sarcomas present with progressive disease, with dire long-term survival rates despite aggressive therapy. In stark contrast to many solid tumors, the prognosis for these cancers has not considerably improved in the past decade. The standard treatment includes a combination of surgery and high-dose chemotherapy, usually with debilitating effects on the short-term and long-term quality of life, as well as a high risk of relapse [5]. Therefore, malignant bone tumors in pediatric and adolescent populations are one of the pediatric cancers that require new therapeutic options.

By harnessing the power of the immune system against cancer in the form of immunotherapy, the survival rates have been dramatically improved in certain cancer types. Especially, immune checkpoint inhibitors (ICI) in solid tumors that target PD-1/PD-L1 and/or CTLA-4 to release the brakes on the anti-tumor T cells yielded effective responses in solid cancers [6]. Unfortunately, pediatric sarcomas, including bone malignancies, have been refractory to these game-changing immunotherapeutic approaches [7]. Therefore, uncovering the molecular players that mediate immune evasion and immunotherapy resistance in pediatric bone sarcoma is pivotal in designing strategies to realize the potential of immunotherapy in these cancers.

Natural killer (NK) cells are immune effectors with tumorlytic activity. These innate effector cells are a heterogenous group of lymphocytes that both exhibit perforin/granzyme B-based cytolytic activity and secrete cytokines that activate the adaptive arm of the immune system. NK-cell adoptive cell therapy (ACT) confers numerous advantages over T-cell ACT, including a lack of major histocompatibility complex (MHC) restriction, no need for prior sensitization, the activation of CD8+ T cells by inflammatory cytokines and a good safety profile with highly reduced graft versus host disease (GvHD) risk [8]. Therefore, NK cells have the potential to be an accessible, safe and feasible immunotherapy approach in hard-to-treat pediatric cancers such as bone sarcomas. There are currently numerous preclinical and clinical studies that are testing adoptive NK-cell therapy in various types of sarcomas. The approaches used in these studies differ in the source of NK cells, the interleukins used and the combinatorial regimen [9]. The reported efficacies have so far been moderate and of short duration due to problems with homing, in vivo persistence, lack of expansion and immunosuppressive tumor microenvironment (TME) conditions [9]. To overcome the current problems with NK cell immunotherapy, there are concerted research efforts in engineering NK cells such as the generation of chimeric antigen receptor (CAR)-NK cells and the use of cytokine combinations to generate memory-like NK cells with enhanced cytotoxic capacity. Primary sarcomas and sarcoma cell lines have been shown to be one of the most vulnerable tumor types to spontaneous NK-cell cytotoxicity; therefore, advances in NK cell immunotherapy have important implications for treating pediatric sarcomas, including bone malignancies [9,10].

In the following sections, we will review the current state of the treatment for pediatric bone malignancies, the long-term effects of these treatments on bone development and growth, the immune evasive strategies of these cancers that jeopardize the immunotherapy efficacy, and the various current NK-cell immunotherapy-based approaches that are being studied in primary bone malignancies by focusing only on EWS and OS, given their prevalence in pediatric populations.

## 2. Primary Pediatric Bone Sarcomas

Primary malignant bone tumors constitute 5–6% of all childhood neoplasms [11]. Malignant bone tumors comprise a highly aggressive group of tumors that have a strong tendency to metastasize. Their current clinical management consists of a multidisciplinary treatment that combines surgery, chemotherapy and sometimes radiotherapy [4]. With advancements in surgical techniques, which allow conservative surgeries to be performed, and advances in chemotherapy, the survival and quality of life of these patients have improved substantially. In recent years, however, the survival rate has stagnated [12]. It is therefore essential to study the etiology and pathogenesis of these tumors to acquire the knowledge for devising targeted, safe, and more effective treatment options.

### 2.1. Epidemiology

OS is the most common primary bone tumor across all age groups, especially in children and young adults, with an annual incidence of 3–4 patients per million, and it represents the eighth most common neoplasm in childhood [13]. The second most common bone tumor in children and young adults is EWS while, in adults, chondrosarcoma ranks as the second [11]. There are a number of differences in clinical presentation and progression between OS and EWS (Table 1).

OS is more frequent in men than in women. It is also more frequent in the child population, with two peaks throughout life. The first peak occurs between 10 and 14 years of age and the second one occurs in patients older than 65 years [14]. In the United States, where there are more reported data, the incidence of OS is higher among African and African-American populations, along with Asian and Pacific Islander populations, compared with Hispanic and white populations [15]. This trend is maintained in southern European countries, especially Italy, where more studies have been conducted [16]. Compared with European countries, in African countries such as Uganda and Sudan a higher OS incidence has been reported [17]. It is also worth noting the high number of rebound cases in the Philippines and Ecuador (rates of 11.4 and 8.2, respectively) [18].

The EWS family of tumors constitute the second most common primary bone tumor in children and represent 40–50% of pediatric malignant bone tumors [19]. These tumors are more common in adolescents and young adults, with a peak incidence at 15 years of age [20]. Similarly to OS, EWS bone tumors are more frequent in male individuals than in females, with a 1.5:1 male to female ratio (Table 1). However, in contrast with OS, EWS is observed in different cohorts around the world and is more prevalent in European populations (1.5 cases per million children and young adults per year) than in African (0.8 per million children and young adults per year) or Asian (0.2 per million children and young adults per year) populations [21].

### 2.2. Pathogenesis

The exact pathogenesis of OS remains unknown, but the relationship between OS and bone growth during childhood and puberty has been studied. The first incidence peak of OS corresponds to the period of fastest bone growth; the endocrine system has a major influence during this period and, therefore, it might also potentially affect OS formation [22]. Examining the process of long bone growth can shed light on the possible connections between OS and bone growth. There are two types of long bone growth; longitudinal and radial growth.

Longitudinal growth occurs in the growth plate or physis, which consists of various layers. During bone growth, a sequential process of cell proliferation and hypertrophy occurs at the physis, followed by synthesis and mineralization of the extracellular matrix (ECM), vascular invasion and then apoptosis, which culminates in the replacement of the cartilage by bone tissue [23]. As a result of cell proliferation and hypertrophy, chondroblasts produce cartilage matrix that is deposited beneath the growth plate. Subsequently, bone-marrow-derived cells differentiate into osteoblasts, which in turn can replace the deposited cartilage tissue, produce osteoid and join the Haversian system along with the osteoclasts. The matrix is mineralized with the deposition of calcium and phosphate to form bone. Other osteoblasts undergo a programmed cell death process [24]. Osteoblast differentiation is regulated by members of the transforming growth factor-β (TGF-β) superfamily, especially by bone morphogenic proteins (BMPs) [25,26].

Radial growth consists of the direct apposition of osteoblasts on the periosteal surface and resorption by osteoclasts on the endosteal surface [27].

These alterations during bone growth might facilitate uncontrolled cell proliferation along with aberrant cell dedifferentiation giving rise to OS.

Two hypotheses have been posited for the origin of OS formation. One of them is based on the mesenchymal stem cells (MSC) and the other one is based on the osteoblasts [28,29]. However, it is likely that both cell types contribute to the onset of OS.

The MSC-based theory states that OS forms due to mutations in the progenitors that lead to an error in the cellular differentiation of the osteoblasts. According to this theory, OS is caused by an alteration in the normal bone formation process, giving rise to a malignant cellular transformation accompanied by an accumulation of chromosomal instability associated with mutations.

Proponents of the osteoblast-centered theory, however, support that the aberration occurs at a later stage of MSC differentiation, given that osteoblasts from patients with OS are still tumorigenic [30].

In addition to the progenitor cells, there are several soluble factors involved in bone growth. Two of these soluble factors are potentially involved in tumor pathogenesis; insulin-like growth factors (IGF) and growth hormone (GH) [31]. GH stimulates bone growth by either acting directly on bone tissue or indirectly via mediators such as IGFs [32]. IGF-1 is the most abundant growth factor in bone and is the mediator of GH’s anabolic effects. IGF-1 can be derived from the peripheral circulation or synthesized in the growth plate by MSCs and osteoblasts in response to GH [33]. IGF-1 levels increase throughout growth and reach their peak at puberty in response to GH, and from thereon IGF-1 levels decrease in parallel with the incidence of OS [34]. Animal tumor models show that IGF-1 is involved in OS pathogenesis and metastatic behavior, although this relationship has not been clearly established [35]. Several proteins mediate the regulation of the IGF-1 signaling pathway. There are six high-affinity binding proteins (IGFBPs) and five low-affinity IGFBP-related proteins (IGFBP-rP). The IGF-1 signaling pathway is attenuated by binding to these proteins in a negative feedback loop as they are stimulated by IGF-1 and, in turn, inhibit the antiapoptotic and mitogenic activity of IGF-1 [36]. This autoregulation mechanism is believed to be disrupted in OS, given that there is a loss of all IGFBPs and IGFBP-rPs compared with healthy bone cells. Therefore, OS cells become self-sufficient by maintaining IGF-1 activity despite IGFBP downregulation [37].

### 2.3. Treatment

Although the mainstay of OS treatment is surgery, which provides good local control of the disease, these cancers tend to spread in the form of micrometastases [38]. Thanks to advances in oncological surgical techniques, procedures that allow for limb salvage are usually performed in more than 85% of cases [38]. The tumor, the biopsy path and the tumor margins need to be completely resected by extensive or radical resections followed by subsequent individualized reconstruction, taking into account the patient’s personal, family, functional and social environment. When limb salvage surgery is not feasible, a mutilating surgery is performed [39].

Neoadjuvant therapy in OS has several functions, including the elimination of microscopic disease, tumor volume reduction, and as a prognostic factor by evaluating the therapy induced necrosis percentage [40]. It has been established that a good treatment response by OS is with percentages of necrosis greater than 90%, and the survival rate is 75% for these patients versus 45% for patients with a poorer response [41]. These rates are much higher than those previously reported given that, before the advent of chemotherapy, 80% of patients with localized disease died within 2 years of the diagnosis as a consequence of metastasis [42].

**Figure 1:**
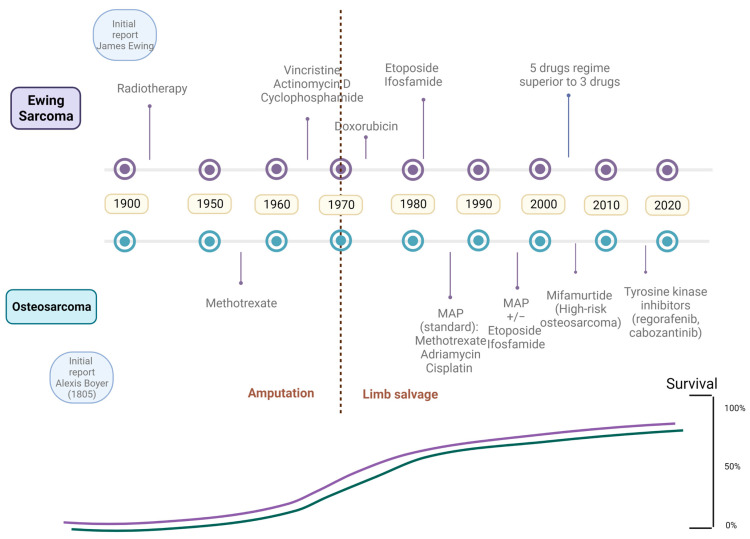
The evolution of standard treatment in Ewing sarcoma and osteosarcoma over time. The associated change in survival is shown in the bottom panel. Purple [top line]: Ewing sarcoma; blue [bottom line]: osteosarcoma [5,20,38,44].

Early treatment regimens for OS were based on the combination of several agents including bleomycin, cyclophosphamide and actinomycin (Figure 1) [43]. However, over the years, these regimens have been modified with development of new chemotherapy drugs and a better understanding of the disease. Agents such as methotrexate, doxorubicin and cisplatin have gradually been introduced into the treatment regimen.

A multicenter randomized study in 1982, high doses of methotrexate along with doxorubicin, and cisplatin (MAP therapy), were administered as postoperative adjuvant therapy [44]. At 2 years posttreatment, the group who underwent the therapy had a 66% disease-free survival rate compared with 17% for the control group. Based on this finding, the treatment of OS was standardized to neoadjuvant chemotherapy with MAP, followed by surgery, and then adjuvant chemotherapy consisting of several cycles of these drugs. Subsequently, various attempts were made to limit the use of cisplatin and doxorubicin due to their long-term adverse effects, and to replace them with ifosfamide or etoposide [45]. The largest randomized OS study to date, the European and American Osteosarcoma Study (EURAMOS) study, was conducted between 2005 and 2016 and involved over 2000 patients from 17 countries with high grade OS. The survival rates recorded in this study are similar to those in previous smaller studies [13]. The results from the study have also shown that adding ifosfamide and etoposide to post surgery MAP therapy in patients that had a poor response to preoperative chemotherapy did not improve the survival rates. Adding IFN-α-2b to post surgery MAP therapy for patients with good response to induction chemotherapy also did not improve the prognosis [46].

In the case of disseminated disease, the same regimen is followed as with localized OS, but the therapeutic approach also includes the removal of metastatic foci, if possible, which are mainly located in the lungs. Additionally, etoposide and ifosfamide are also employed more routinely [47]. Despite these efforts, overall survival remains quite low, with a long-term survival rate of 20% [45].

Radiotherapy is the main difference between OS and EWS in terms of clinical management (Figure 1). The first EWS treatment regimens consisted of a combination of various cytotoxic agents such as vincristine, dactinomycin and cyclophosphamide (VAC), combined with doxorubicin [48]. Several clinical trials have subsequently been launched; Grier et al., studied the addition of other cytostatic agents such as ifosfamide and etoposide to the standard EWS protocol, observing an added benefit in localized disease, but not in metastatic disease [49].

Currently, the standard protocol consists of neoadjuvant chemotherapy followed by local treatment with surgery and/or radiotherapy. The treatment choice depends on the location and the size of the tumor, and the age of the patient. After local treatment, adjuvant chemotherapy is administered to remove residual disease.

After local treatment, patients with poor prognostic factors and high-risk EWS might benefit from myeloablative chemotherapy with busulfan and melphalan for conditioning therapy, followed by an autologous peripheral cell transplantation, as observed by the Spanish group led by Miguel A. Diaz [50].

### 2.4. The Effects of Sarcoma Treatments on Bone Growth

There are several factors that influence the bone mineral density (BMD) of patients with cancer; intrinsic factors, such as the tumor type and location, and extrinsic factors, such as the administered treatments.

During cancer therapy, patients experience a lack of appetite, anorexia, immobility and reduced exercise, all of which predispose them to a state of cachexia, generating metabolic changes that affect various body systems, especially bone growth. Additionally, these patients also frequently experience vitamin D deficiency. All these factors give rise to a lower BMD during growth, which weakens the bones and increases the fracture risk [51].

#### 2.4.1. Chemotherapy

**Figure 2:**
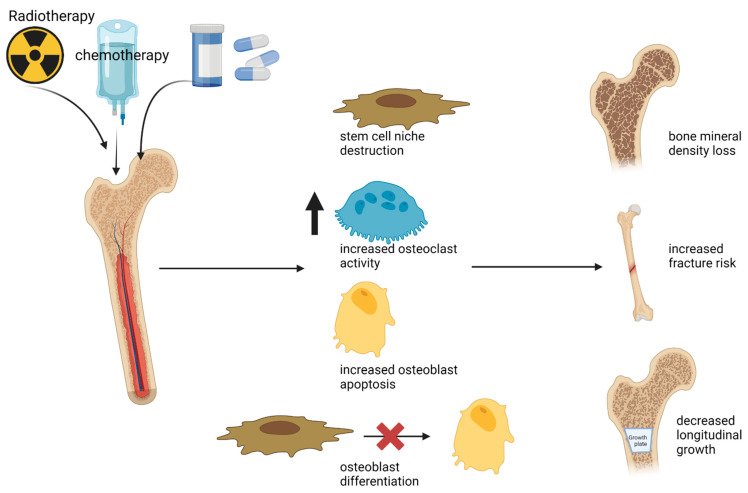
The effects of sarcoma therapy on bones. Both chemotherapy and radiotherapy have negative effects on bone growth and BMD. These therapies can obviate the stem cell niches required for BMD maintenance, as well as inhibit the differentiation of stem cells into osteoblasts (inhibition is shown by the red cross in the figure). The therapies can also increase bone resorption by inducing osteoclast activity and osteoblast apoptosis, leading to decreased BMD, increased fracture risk and decreased longitudinal bone growth.

Chemotherapy agents commonly employed in several oncological mechanisms have a negative effect on the BMD (Figure 2).

Methotrexate is a folic acid analog employed for treating inflammatory diseases (such as rheumatoid arthritis) at low doses and is used at high doses for treating neoplasms. Methotrexate is the most widely used chemotherapy agent for treating pediatric sarcomas and hematological malignancies [52]. The drug also has secondary adverse effects at the bone level, such as osteopathy, bone pain and an increased risk of fracture.

Methotrexate causes growth plate dysfunction, reducing the proliferation of chondrocytes and the amount of type II collagen, leading to apoptosis, which affects bone elongation [52]. Methotrexate also reduces the density of osteoblasts on the surface of the trabecular bone and acts on MSCs, producing alterations in their differentiation; methotrexate causes MSC exhaustion or suppression with a resultant decrease in osteoblasts [53]. A significant increase in bone marrow adiposity has also been found in these patients. A study showed that methotrexate administered to rats produces an imbalance in bone remodeling in favor of resorption, because it increases the number of osteoclasts [51].

Other chemotherapy agents associated with decreased bone mineralization that have been studied in vitro include doxorubicin, etoposide, vincristine, cyclosporine, tacrolimus, cyclophosphamide and 6-mercaptopurine. The disadvantage of these studies is that the chemotherapy agents have not been studied separately [51].

#### 2.4.2. Radiotherapy

Treatment with ionizing radiation causes bone injury that can lead to bone insufficiency and low BMD. These effects were observed when women who underwent pelvic irradiation for treating different types of carcinomas had a hip fracture risk three times higher than that of women that didn’t receive radiation therapy [54].

Radiotherapy is based on the liberation of free radicals and reactive oxygen species, which leads to cellular damage, such as DNA damage [55]. Moreover, this damage is repaired in physiological conditions by stem cells, which are also destroyed by radiotherapy (Figure 2). Cao X et al., demonstrated that irradiating the mouse femur destroys the vascularization of the stem cell niches [56].

Sakurai T et al., studied the effect of radiation on osteoblast differentiation and suggested that radiotherapy at therapeutic doses modifies the cell cycle that affects the number of osteoblasts and increase bone fragility [57]. Furthermore, the irradiated areas showed higher apoptosis and cell cycle arrest that led to reduced numbers of osteoblasts [58]. The apoptotic effect can further increase with the addition of chemotherapy.

Radiation therapy also causes temporary increases in the expression of specific genes involved in bone resorption. It also increases the liberation of proinflammatory cytokines and osteoclastic ligands that stimulate osteoclastogenesis in the presence of RANKL or TNF-α [59,60]. This activation of osteoclastogenesis leads to acute cancellous bone loss proportionate to the radiation dose [61].

Due to the nature of the treatment modalities, primary bone sarcomas cause long-lasting effects on bone growth and development, even when mutilating surgery is avoided. Therefore, safer treatment approaches are required against these cancers. As immunotherapy has become the fourth pillar of cancer treatment, efforts to understand the barriers to the success for immunotherapy can help in designing safe and effective treatments.

## 3. Barriers to Immune Responses and Immunotherapy in Pediatric Bone Sarcomas

**Figure 3:**
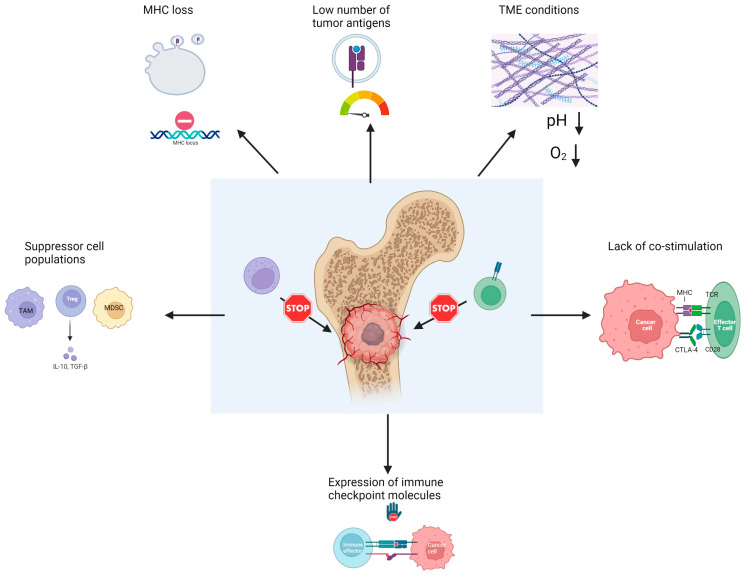
Mechanisms that mediate the immune exclusion in osteosarcoma and Ewing sarcoma. Immune responses against primary bone malignant tumors are inhibited at multiple levels. MHC loss that is primarily mediated by reduced expression and shedding of MHC molecules decrease the T-cell recognition of the tumor cells. Immune recognition is also hampered by the low number of tumor-associated antigens (TAAs) and central and peripheral tolerance mechanisms that prevent immune cells from recognizing self-antigens on the tumor cells. Co-stimulation required for T-cell activation is also hampered by CTLA-4 upregulation. Dense, hypoxic and acidic TME conditions prevent immune effector infiltration and activation against the bone sarcoma cells. Furthermore, suppressive immune cell populations, which are either recruited to the tumor site or differentiate into a regulatory phenotype in TME, can cause anergy or death of anti-tumor immune effectors. Various immune checkpoint molecules expressed by the tumor cells also deliver inhibitory signals against immune cell activation (MHC: major histocompatibility complex; TAM: tumor-associated macrophages; Treg: regulatory T cells; MDSC: myeloid-derived suppressor cells).

Many pediatric solid cancers, including bone malignancies are generally ‘cold’ tumors that do not harbor many mutations and many tumor-infiltrating lymphocytes (TIL), with poor responses to immunotherapies. The presence of several immune effectors in EWS and OS has been associated with the prognosis. The tumor’s evasion of the host’s immune response is one of the hallmarks of cancer [62]. The immune evasion strategies of these tumors can present at each phase of an immune response, from target recognition to the suppression of the immune effector functions (Figure 3). These evasion mechanisms also shape the tumor’s immune environment and fate. For example, when RNA-seq data were employed in pediatric OS to evaluate the immunologic constant of rejection (ICR), the ICR score positively correlated with improved survival [63]. ICR is comprised of 20 genes that collectively show an active Th1 and tumorlytic response, such as IFN-γ and granzyme B expression. The association between the OS prognosis and ICR underscores the fundamental influence of the immune response on the tumor’s fate. A study that compared the immune infiltrates between OS and EWS by using a single-cell RNA-seq analysis of the patients’ (aged 13–19 years) blood and tumor samples, as well as using publicly available genomic databases, showed that the immune infiltrates and their frequencies differed between these two primary bone sarcomas, as well as between relapsed and primary tumors within each type, demonstrating that the tumors’ immune landscape is shaped by the dynamic and continuous interaction between the tumors and the immune system [64]. It is of paramount importance, therefore, to understand the immune–tumor interactions and the barriers to an effective immune response in order to tailor effective immunotherapies for these malignancies.

### 3.1. Low Number of Tumor-Associated Antigens

The immune recognition of tumors is intrinsically challenging because there are both central and peripheral tolerance mechanisms in place that prevent immune effectors from recognizing self-antigens. Given that cancer arises from the body’s own cells, the number of TAAs that are specific to a tumor is usually low. These tolerance mechanisms limit the repertoire of T and B cells that can respond to the tumor cells. Overexpressed genes, re-expression of genes from fetal life, mutations, and aberrant post-translational modification make up the majority of TAA sources [65]. TAAs can be used both for activating B and T cells, and for actively directing cytokines, toxins, chemotherapeutic and radiolabeled molecules to the tumor site.

The lack of TAAs is especially prevalent in pediatric cancer. Unlike most adult cancers, pediatric bone malignancies (especially EWS) do not have a high mutation rate [66]. Although the factors that predict the success of ICI have not been clearly elucidated, “hot” tumors that harbor more mutations are generally more likely to respond to ICI [67]. EWS is characterized by a pathognomonic chromosomal translocation that results in a chimeric protein generated from the fusion of the EWS and FLI1 genes; however, this protein does not have enzymatic activity that is druggable, although CRISPR-based techniques are being developed to edit out this fused gene [68]. Regardless, EWS has one of the lowest somatic mutation rates among tumors and, therefore, the types of antigens that can be targeted by immunotherapy are highly limited.

One of the TAAs being targeted in EWS is GD2, a disialoganglioside. Unlike many other gangliosides, this carbohydrate-containing sphingolipid has restricted expression in normal tissues and is overexpressed in many solid tumors, including EWS [69]. GD2 expression has been shown to enhance the proliferative and metastatic capacity of tumors. Anti-GD2 monoclonal antibodies (mAbs) have been approved by the Food and Drug Administration (FDA) and European Medicines Agency (EMA) for use in high-risk neuroblastoma in pediatric patients. However, problems with peripheral neurotoxicity limit the use of this approach [70]. A study with three cases of metastatic EWS showed that the use of the GD-2 monoclonal antibody in combination with chemotherapy as first-line therapy led to complete remission [71]. In addition to targeting these TAAs with mAb, GD-2-specific CAR T cells have been designed. A preclinical study showed that combining anti-GD-2 CAR T-cell therapy with human hepatocyte growth factor inhibition prevented tumor growth and metastasis in a xenograft EWS mice model [72]. Although CAR-T-cell therapy has significantly enhanced survival for hematological malignancies, it has had limited efficacy in solid tumors due to numerous factors, including the heterogeneous expression of target antigen, tumor homing problems and suppressive TME. Nonetheless, there are clinical trials that are currently recruiting GD-2+ pediatric and adult patients for GD-2 CAR-T cell therapies, including a trial that is using a 4th-generation CAR-T cell with a safety switch (inducible suicide caspase 9 gene) (NCT03356782). Other antigenic targets in EWS include ROR1, stem cell marker CD133 and IGF1R [73,74].

B7-H3 (CD276) is an immune checkpoint molecule with an, as of yet, unidentified receptor that has structural similarities to PD-L1. B7-H3 is aberrantly expressed in certain solid tumors, including EWS, with approximately 90% of pediatric EWS cases presenting positive B7-H3 expression [75]. A study that tested an antibody drug conjugate containing B7-H3 Ab with pyrrolobenzodiazepine in pediatric EWS-patient-derived xenografts showed that the conjugate decreased the tumor volume in all the generated xenografts [76]. There are also ongoing clinical trials testing the safety of various B7-H3 CAR T-cell constructs in pediatric solid tumors (NCT04897321, NCT04483778).

Compared with EWS, OS has a higher mutational burden but still has one of the lowest mutations per megabase (0.38) [5]. Instead of point mutations, copy number alterations are frequently observed in OS. The mutations frequently observed in OS are in prototypical tumor-suppressor gene, p53 and in protooncogene retinoblastoma [4].

Compared to EWS, OS is infiltrated with more immune cells [64]. OS shares certain common TAAs with EWS, such as B7-H3, IGFR1 and GD-2. In the abovementioned study with B7-H3 antibody-drug conjugate, one of the OS xenografts showed no objective response, underscoring the presence of immune evasion mechanisms [76]. An immunohistochemical analysis of patient samples showed that B7-H3 was the most highly expressed immune checkpoint molecule in advanced OS; however, the study included both pediatric and adult cases [77]. In addition to B7-H3, GD-2 is expressed frequently in OS, and anti-GD-2 antibodies such as dinutuximab (a chimeric IgG1 monoclonal antibody) can be used in OS [78]. A humanized anti-GD-2 antibody in combination with granulocyte macrophage colony-stimulating factor are currently being tested in a phase II clinical trial for adult and pediatric patients with recurrent OS (NCT02502786) A preclinical study using cell-line and patient-derived xenografts has shown that T cells that are ex vivo “armed” with anti-GD2 and anti-CD3 bispecific antibodies significantly slowed tumor growth and enhanced survival in mice [79]. This approach caused no significant toxicity in the animal model, underscoring the need for the fine-tuning or manipulation of current immunotherapeutic approaches to extend their uses to more types of cancer, including malignant bone sarcomas. Other TAAs that can be targeted in OS include HER-2 and ROR-1 [5].

Given that metastatic disease, especially in the lungs, is the leading cause of mortality in these types of cancer, immunological targeting of the molecules associated with metastasis is a potential strategy to improve survival. For example, it has been suggested that alpha-4 integrin sustains the survival of OS cells during their spread from the primary tumor site [80]. Natalizumab, a monoclonal antibody specific to alpha-4 integrin, is being tested in a phase I/II clinical trial in patients between the ages of 5 to 30 with recurrent refractory pulmonary metastatic OS (NCT03811886). A meta-analysis confirmed another molecule associated with metastasis in OS: CXCR4 [81]. Our laboratory has previously shown in a rhabdomyosarcoma animal model that targeting CXCR4 with a monoclonal antibody in combination with activated and expanded NK-cell therapy prevented lung metastasis and tumor implantation [82]. Overall, not only TAAs but also metastasis-associated molecules could act as effective targets in immunotherapy.

In addition to the already existing TAAs, tumor antigenicity can be increased by various means. For example, chemotherapy and radiotherapy can contribute to the formation of neoantigens by causing mutations in the tumor cells. Furthermore, these interventions can cause immunological cell death (ICD). ICD refers to the stimulation of immune responses by cell death. ICD induction is usually achieved through inducing the expression of damage-associated molecular patterns (DAMPs) and creating a proinflammatory environment through cytokines. DAMPs such as adenosine 5′-triphosphate (ATP) and calreticulin are endogenous molecules that are released from damaged and dying cells to signal “danger” or threat to the immune cells. DAMP generation can be prompted by cell damage due to chemotherapy and radiotherapy. By binding to the pattern recognition receptors on dendritic cells (DCs), DAMPs can act as maturation stimuli for the cells, which can then present antigens to T cells to initiate T-effector functions [83]. Abscopal effect serves as a good example of ICD. In abscopal effect, tumor cells that are not located at the region targeted by the radiotherapy are cleared by the immune cells that have been activated by the therapy-induced cell damage. A study showed that when radiofrequency ablation was combined with localized OK-432 (streptococcus group A mixture) administration in a rat OS model, the number of distant tumors decreased significantly, demonstrating the abscopal effect of this local therapy [84].

Besides secreted molecules, the upregulation of stress molecules, such as MICA/B, by stress induced by the therapy can engage the activating NK-cell receptors, such as NK group 2 member D (NKG2D), to stimulate a cytolytic response by NK cells. NK-cell activation occurs when there is a “net” activating signal, upon adding the cumulative effects of the various inhibitory and activating receptors. Therefore, inducing stress responses in tumor cells can directly contribute to the effector functions of NK cells.

### 3.2. MHC Downregulation

As previously mentioned, the immunotherapeutic targeting of tumors by antibodies and/or T cells is hampered by the scarcity of TAAs. Another problem is the active immune evasion by the tumor cells. One of the strategies the tumor employs to escape T-cell recognition is the downregulation of MHC I expression. Both EWS and OS have a very low MHC class I expression [5]. Fortunately, NK cells do not depend on antigen presentation of MHC but rather on the engagement of multiple receptors. NK cells also actively counteract this evasion mechanism by killing cells that do not express MHC cells (missing self), given that inhibitory receptors such as CD94/NKG2A heterodimers bind to HLA molecules [85]. As will be discussed in the later sections of this manuscript, this HLA-independent activation of NK cells represents an important advantage over T-cell-centered immunotherapies.

The tumor cells achieve MHC downregulation mainly through epigenetic regulation [86]. Therefore, drugs that modify the tumor cells’ epigenetic programming, such as histone deacetylase inhibitors and DNA methyltransferase inhibitors, can increase tumor antigenicity by reinducing the expression of the genes involved in antigen processing and the presentation pathway. For example, enhancer of zeste homologue 2 (EZH2) is a protein involved in epigenetically silencing genes, including genes coding for MHC molecules. EZH2 expression in OS is associated with worse prognosis, which could be partially due to MHC downregulation [87]. A study on head and neck squamous cell carcinoma showed that EZH2 inhibition increased MHC expression in the cell lines from this tumor type [88].

In EWS, EWSR1-FLI1, the signature fusion protein of EWS, can lead to chromatin remodeling that activates the regions of the genome that were previously silenced, by superenhancers [89]. Therefore, reverting epigenetic programming in EWS can have therapeutic implications both in and beyond immunotherapy. In NK cells, it has been shown that EZH2 silencing or inhibition enhances NK-cell function both for murine and human NK cells, yet the mechanism is probably not through MHC upregulation but by the upregulation of the “MHC-equivalent” for NK cells, NKG2D [90]. The silencing of the pregnancy-associated plasma protein-A (PAPP-A) that promotes IGF secretion has also been shown to increase antigen presentation proteins, and EWS tumors with a lower PAPP-A expression have more RNA for immune response pathways [66].

Exosomes are lipid-bilayer-enclosed extracellular vesicles that range between 30 and 150 nm and mediate intercellular communication by transferring bioactive molecules such as RNA, microRNA, lipids and proteins between the cells [91]. Another one of the tumors’ immune evasion strategies is the shedding of MHC molecules and cognate antigens in extracellular vesicles, which divert the effector T cells from the tumor cells.

### 3.3. Decreased Costimulation

T cells require antigen presentation in MHCs (signal 1), as well as costimulatory signals such as CD28 (signal 2), to become activated. The upregulation of the inhibitory signals such as CTLA-4, instead of costimulatory ones, can lead to T-cell anergy. A pooled analysis showed that CTLA-4 +49G>A polymorphism was associated with malignant bone tumor risk, including in OS and EWS [92]. Furthermore, CTLA-4 had a higher expression in T cells from pediatric patients with OS than in healthy controls [93]. CTLA-4-blocking mAbs (e.g., ipilimumab) are one of the main ICIs; however, a phase 1 study that included pediatric patients with recurrent or progressive sarcoma failed to show any objective response, despite increased CD4+ T-cell counts [94]. One of the possible reasons for this failure is the presence of multiple and overlapping immune checkpoint molecules other than CTLA-4. These molecules could be B7-H3 (as mentioned earlier) and lymphocyte-activation gene 3 (Lag-3) that binds to MHC class II molecules. In metastatic OS, Lag-3 expression at the interface between normal tissue and pulmonary metastasis as detected by immunohistochemistry, is associated with poorer progression-free survival [95].

### 3.4. Immunosuppressive TME Conditions

In addition to the discussed hurdles for immune recognition in malignant osseous tumors, even when immune effectors can get activated there are still challenges remaining for reaching to the tumor site and maintaining the activation state. The TME is of special concern because it also has direct implications for the success of adoptive cell therapies, given that if the infused cells cannot infiltrate the tumor site and/or stay active, the treatment approach is essentially nullified. The TME of bone malignancies contains multiple cell types including stromal cells such as fibroblasts, immune cells, osteoblasts, osteoclasts, vascular cells and the non-cellular physical part of the tumor, the ECM. The osteoid ECM actively enables tumor proliferation and dissemination [96]. In the tumor, ECM can change cell adhesion, act as a reservoir for growth factors (e.g., stem cell factor), block immune infiltration, provide a niche for cancer stem cells, and modulate cell differentiation [97]. Tumor-associated ECM, which is denser than normal ECM, can act as a physical barrier against drugs and immune effectors reaching the tumor, thereby directly contributing to the therapeutic resistance of tumors [98].

Collagen I is the primary protein in the dynamic bone TME. The deposition of fibrillary collagen that is associated with cancer fibrosis is mainly mediated through matrix glycoproteins, such as fibrin and fibronectin [99]. The immunohistochemical staining of OS samples has shown that fibronectin expression in the tumors is associated with reduced response to chemotherapy and overall survival [100]. In EWS, a tumor type where stromal cells are not dominant, LC-tandem mass spectrometry of two EWS cell lines has shown that fibronectin is one of the primary components of the EWS secretome [101]. In general, the thick ECM of these tumors acts as a barrier against immune infiltration of the tumor and promotes the immune exclusion of the tumors.

The immune exclusion of the tumors can also be mediated by the tumor vasculature, mainly by downregulation of adhesion molecules to inhibit lymphocyte homing or by expressing death receptors, such as FasL, to promote death of immune effectors. To infiltrate the tumors, activated immune effectors need to bind to cell adhesion molecules on the vasculature, such as intercellular adhesion molecule 1 (ICAM-I). A study on the RNA sequencing data of 93 patients with OS divided the patients into 2 groups based on the expression of angiogenesis-related genes with prognostic significance, showing that the cluster with better survival expressed more adhesion molecules (such as VCAM-1 and ICAM-1) in the vasculature [102]. The study also showed that this cluster with higher adhesion molecules had a concomitant higher infiltration by immune cells such as CD8 + T cells, B cells, and regulatory T cells (Tregs). However, in both OS and EWS, ICAM-1 expression on the tumor cells has been shown to promote cell motility and metastasis [103]. These findings underscore the fact that the effects of molecules can depend on their spatial and temporal expression and on the cell source. Targeting a single molecule in cancer therapy is therefore highly likely to result in heterogeneous responses, which also demonstrates that improved understanding of tumor biology is needed to design smart therapies that incorporate the complex interactions among tumor components.

Vascular endothelial growth factor (VEGF)-A promotes tumor-associated angiogenesis, and the monoclonal antibody that targets VEGF, bevacizumab, can potentially target both tumor-promoting angiogenesis and enhance immunotherapy. However, a phase II study with 32 patients with OS (with a median age of 12.8 years) found that adding bevacizumab to standard OS chemotherapy produced no clinical benefit [104]. However, a retrospective analysis of 39 patients (with a median age of 15 years) who were treated with bevacizumab combined with sorafenib and cyclophosphamide showed that a total of 16 of the patients with bone sarcoma (EWS and OS) had at least stable disease [105]. Although these results have yet to be confirmed by randomized controlled trials, the authors suggest that bevacizumab is a candidate for addition to upfront or maintenance regimens in bone sarcomas.

TME is also characterized by its hypoxic nature, which is caused by disorganized tumor vascularization and rapid cell proliferation. Hypoxic TME recruits immunosuppressive immune populations and promotes M2 macrophage differentiation [106]. Tumors also upregulate several proteins that aid in their survival under hypoxia, such as hypoxia-inducible factor 1 (HIF-1). A retrospective analysis of 30 pediatric patients who were diagnosed with high-grade OS employed immunohistochemical staining to show that HIF-1α expression was correlated with the presence of CD68+ macrophages and poorer disease outcomes [107]. In the hypoxic environment, tumor cells mainly perform glycolytic respiration, which leads to the accumulation of lactate, and acidosis that, in turn, inhibit NK and T-cell function [106,108]. A study on bone and soft-tissue EWS stratified patients into high and low-risk groups based on differentially expressed hypoxia-related genes. The high-risk group had higher expression of T cell co-inhibition related molecules [109]. This finding strongly suggests a link between hypoxia and immunosuppression in EWS.

### 3.5. Immunosuppresive Cell Populations

In addition to the mechanical and metabolic barriers posed by the TME, soluble factors, such as IL-10 and TGF-β, present in the TME can affect immune cell differentiation and function to promote tumor survival. These inhibitory cytokines can be secreted by tumor cells themselves and by suppressive immune cells, such as tumor-associated macrophages (TAMs), myeloid-derived suppressor cells (MDSC) and Tregs.

Among the immunosuppressive immune populations, TAMs are especially important for pediatric EWS and OS given that they are the most abundant immune population in the TME, with OS being more heavily infiltrated by monocytes than EWS [5,64,110]. In the TME, macrophages can transform into tumor-supporting TAMs (M2 macrophages) [111]. These cells secrete molecules such as IL-10, VEGF and TGF-β, which promote angiogenesis and inhibit the formation of an effective immune response. Conversely, macrophages with the M1 phenotype recruit cytotoxic T cells and Th1 cells into tumor tissue, thereby aiding the anti-tumor immune response. The presence of CD14 + CD16+ macrophages in OS has been positively associated with better overall survival [64]. However, the impact of macrophage density in OS appears to depend on the cancer stage and treatment phase, as the presence of higher density of macrophages before chemotherapy is associated with less metastatic events; after chemotherapy, however, there is a positive association between higher density of macrophages and metastasis [112].

Chemotherapy can change the tumors’ immune landscape and can create selective pressure towards tumor clones that are more immunosuppressive. A study on OS cell lines and patient samples showed that chemotherapy induces a macrophage immune checkpoint molecule, CD47, which inhibits phagocytosis [112]. It has already been demonstrated that myeloid cells, such as macrophages, have subpopulations with differing expression of genes such as complement receptors and therefore differ in their biological roles [64]. Studies that incorporate more than surface markers of the cells, and also study the functional signatures of these cells are therefore needed to explain some of the seemingly contradicting data in the literature. In the study mentioned above that correlated HIF-1α expression with CD68+ cells in OS, it is interesting to note that CD68+ cells are normally taken as the marker of M1 macrophages [107]. Therefore, it is possible that M1 macrophages might have protumoral activities in OS, and M1/M2 dichotomy might need to be revised for this cancer type.

In EWS, on the other hand, a predominance of CD68-positive macrophages is associated with poor survival in humans [113]. In a xenograft murine metastatic EWS model, metastasis was dramatically reduced when macrophages were selectively inhibited, and it was shown that M2 macrophages promote tumor cell extravasation at the site of metastasis [114]. When myeloid signature genes were analyzed from RNA-seq data and were correlated with clinical information available in various databases, it was possible to divide patients with EWS into high- and low-risk groups based on the expression of three myeloid signature genes using a generated formula [115]. It has been shown that the high-risk group had a higher infiltration of M2 macrophages, whereas the low-risk group had a higher infiltration of CD8+ T cells and NK cells.

Various types of monocytes and macrophages found in the body can also contribute to TAM formation. Alveolar macrophages can differentiate into the M2 phenotype in lung metastases of OS as a result of the extracellular vesicles secreted by the tumor cells, and their presence is associated with shorter survival [116]. The differentiation of bone-resident macrophages (osteoclasts) is also increased in OS due to the secretion of RANKL from osteoblasts that bind to the RANK on the cells [117]. To the best of our knowledge, transdifferentiation between osteoclasts and M2 cells in cancer settings has not yet been demonstrated. In addition to their bone reabsorption function, osteoclasts have been shown to induce Treg differentiation to create an immunosuppressive environment [118]. Although we can speculate that osteoclasts play a role in the immune microenvironment of OS and osseous EWS, this concept remains to be clearly elucidated.

MDSCs are another group of suppressive immune cells that help tumors evade immune surveillance. MDSCs have an immature phenotype. They express the myeloid marker, CD33 and arginases, with an ability to suppress T-cell responses [119]. MDSCs can mainly suppress the immune response with cytokines such as IL-4 and arginases. For example, arginase 1 inhibits T-cell activation by depriving L-arginine from the medium. In the absence of L-arginine, T-cell-receptor-(TCR)-mediated activation signals are inhibited, thereby MDSCs can suppress T-cell activation from the initial steps of activation. A murine OS model showed that MDSCs were more abundant in the blood and tumors of mice in an IL-18-dependent manner than in the control group [120]. Only concomitant IL-18 and PD-1 blocking decreased MDSC infiltration of the tumor and increased the anti-tumoral T-cell responses, slowing tumor progression [120]. MDSC staining in OS tissues has also been observed to be inversely associated with the presence of CD8+ T cells [121]. The same study showed that CXCR4, a metastasis-related molecule in OS, is also involved in attracting MDSCs to the murine OS TME by binding to SDF-1 (CXCL12). As with the results obtained with IL-18 and PD-1 co-blocking treatment, combining PD-1 and CXCR4 blocking increased CD8+ T-cell infiltration of the tumors and enhanced the survival of the tumor-bearing mice [121]. When murine xenograft models were constructed using human OS and EWS (soft tissue EWS), MDSC numbers were increased in the blood, spleen and tumors of mice [122]. A third-generation GD2-CAR that was ineffective against the generated xenograft OS model slowed tumor growth and increased survival in mice when CAR-T therapy was combined with MDSC “neutralizing” treatment (administration of all-trans retinoic acid) [122]. These findings cumulatively show the importance of MDSC in the efficacy of immunotherapies in pediatric sarcomas, especially in OS.

The main immunosuppressive immune cells in TME are usually Tregs. When these cells are present in the TME, they prevent T-cell activation and generate T cells that do not respond to antigens (T-cell anergy) [123]. Tregs mediate this suppression by secreting adenosine and cytokines, such as IL-10, or by causing the death of T cells (e.g., by activating death receptors) [123]. In a flow cytometric analysis of the immune cells in the bone marrow of patients with bone EWS (median age of 14 years; range 8–25 years), patients who had primary metastatic disease had higher Treg frequencies than those who had localized disease [124]. In another analysis, Treg frequencies were increased in the peripheral blood of the patients with EWS; however, there was no association with disease outcomes [125]. A study on the pretreatment biopsies from patients with OS (median age of 15.5 years; range 6–70 years) has shown that CD8+/FOXP3+-ratios above 3.0 in immunohistochemical staining at the time of diagnosis were associated with better survival regardless of presence of metastasis at diagnosis and responsiveness to neoadjuvant chemotherapy [126]. In the ICR study mentioned earlier, however, enrichment in Treg transcripts was associated with better survival in OS [63]. It is thus possible that the ratios of effector to suppressor cells could be more informative than just the frequencies of individual cells.

### 3.6. Immune Checkpoint Molecules

The molecules that normally prevent aberrant lymphocyte activation and overaction are frequently upregulated in tumor settings as another immune evasion mechanism in cancer. It is safe to state that ICIs have been the single most groundbreaking immunotherapy approach in solid tumors. Targeting the immune checkpoint molecules CTLA-4 and PD-1/PD-L1 axis through mAbs is the most widespread approach in these therapies. Unlike CTLA-4, which prevents T-cell activation, the PD-1/PD-L1 axis induces the death of the activated lymphocytes, mainly T cells. Expression levels of PD-1/PD-L1 have been suggested as one of the predictors of the response to ICI. However, EWS cells do not normally express these molecules [127]. Combined with the low mutational burden of these tumors, the scarce expression of immune checkpoint molecules can explain, at least partially, the lack of success of ICI in these tumors. OS, as the more immunologically ‘hot’ tumor of the two, has a higher frequency of PD-L1 expression, which has been correlated with the number of TILs and poorer event-free survival [128]. A study on high-grade OS tumor samples has, however, shown by immunohistochemical staining that PD-L1 expression was infrequent (9% of the cases) [77]. A study on tumor biopsy samples collected from patients at three points (diagnosis, resection and from the metastatic mass) have shown that PD-1/PD-L1 expression might differ depending on the specimen of choice [129]. A clinical trial involving young patients with recurrent or refractory solid tumors including EWS and OS, using nivolumab (anti-PD-1) with or without ipilimumab (anti-CTLA4), unfortunately failed to show efficacy in the results (NCT02304458).

Another immune checkpoint molecule investigated in bone sarcomas is T-cell immunoglobulin and mucin domain-containing-3 (TIM-3). TIM-3 is a marker for T-cell exhaustion and an inducer of apoptosis. TIM-3 is expressed more widely than PD-L1 by OS cells, and its expression has been linked to poor overall survival in patients with OS in a study where 55% of the patients were younger than 20 years old [130]. In pediatric EWS, TIM-3 expression is observed frequently (all 10 of the 10 cases) in immunohistochemistry staining of tumor samples [131]. The efficacy of blocking TIM-3 by mAbs remains to be tested in EWS.

CD8+ T cells in OS express multiple immune checkpoint molecules including PD-1, TIM-3, Lag-3 and T-cell immunoglobulin and ITIM domain (TIGIT) [64]. ITIM is another emerging immune checkpoint molecule expressed on T cells and NK cells. The main ligand of TIGIT is CD155, which is also a ligand for the activating NK cell receptor DNAM-1. TIGIT has been proposed to impede anti-tumor responses by impairing T-cell priming, NK and T-cell activation [132]. CD8+ T cells from OS lesions have a high expression of TIGIT, signaling their exhausted state [133]. Tregs in OS also express this molecule, and blocking TIGIT by mAb enhanced the ex vivo killing of OS cell lines by CD3+ T cells isolated from patients with OS [133]. In EWS, on the other hand, CD8 + T cells express galectin-3 (gal3) [64]. Gal3 can induce both T-cell death and TCR downregulation and is upregulated in musculoskeletal tumors [134].

Overall, OS and EWS have thus far been refractory to immunotherapeutic approaches; however, an increased understanding of the immune landscape of these tumors and the use of advanced biotechnological tools to boost the effectiveness of the immune cells could yield effective strategies. NK-cell-based approaches in primary pediatric bone malignancies have yet to be transferred to clinic but, once these approaches are optimized, they have a high chance of being the successful immunotherapy option for these cancers.

## 4. NK Cell-Based Therapies in Pediatric Bone Sarcomas

### 4.1. NK Cells

NK cells are a heterogeneous immune population with varying degrees of cytolytic and cytokine secretion potential. The functional heterogeneity is mirrored in the phenotype of NK subsets, which express a variety of combinations of germline-encoded NK cell receptors at various levels.

Ninety percent of peripheral blood NK cells have a distinct CD56dim CD16+ phenotype, whereas the remaining ten percent consists of CD56bright CD16− NK cells. CD56 (human neural cell adhesion molecule) has been linked to cytolytic functions in NK cells and is not expressed on murine NK cells. CD16 is the low affinity FcγRIII that enables the CD56dim CD16+ subset to kill antibody-opsonized target cells via antibody-dependent cell cytotoxicity (ADCC), and these cells are more granular and cytotoxic than the CD56bright CD16− subgroup [9]. CD56bright CD16− NK cells, on the other hand, are major producers of NK-cell-derived cytokines (such as IFN-γ and TNF-α) and chemokines (such as MIP-1α) and are assumed to be the less mature one of these two NK cell population.

NK cells express a wide array of germline-encoded activating receptors, inhibitory receptors, co-receptors and adhesion molecules that are at the center of the regulation of their activation. Structurally, NK receptors can be grouped into those that belong to the immunoglobulin superfamily and to the C-type lectin superfamily. Killer-cell immunoglobulin-like receptors (KIR) and cytotoxicity receptors (such as NKp30, NKp44 NKp46) belong to the former group, whereas NKG2 receptors belong to the latter. At the clonal level, not all NK cells express the same repertoire of receptors, leading to differing functional sensitivities and potencies9 among NK cells.

Similar to T cells, a highly organized immunological synapse forms at the contact site of the NK cell and target cell. The activating immunological synapse between the NK cell and its susceptible target triggers a series of downstream events that, unless counteracted by the inhibitory receptors, leads to the release of preformed cytolytic granules containing perforin and granzyme B. Upon ligation of inhibitory receptors, however, SHP-1 and SHP-2 are recruited, and they stop the actin polymerization and cytoskeletal rearrangements required for cytolytic granule secretion [135].

### 4.2. NK Cells in Cancer

NK cells have proven to be important players in anti-tumor responses, given that they lyse tumor cells that downregulate MHC class I and express stress-inducible proteins. Studies in mice have shown that there is a positive correlation between NK activity and the clearing of transplanted tumors [136]. Furthermore, a 11-year follow up study in humans revealed that higher NK activity in the peripheral blood positively correlated with a lower risk of developing cancer [137]. A recent meta-analysis that included 56 studies showed that tumor-infiltrating NK cells, as assessed by CD56, NKp30, NKp46 and CD57 markers, are a good prognostic factor for overall survival in solid tumors [138]. The employment of NK cell escape mechanisms by tumors, such as the shedding of soluble NKG2D ligands, shows that NK cells create selective pressure on tumor cells and are important in clearing transformed cells [139].

Based on these findings, it would be expected that therapies that elicit NK responses would be more effective than similar therapies that do not engage NK cells. Researchers have shown in mice that DC vaccines elicited an increased NK response (IFN-γ production and cytotoxicity) that contributed to the anti-tumoral responses [140]. The efficacy of rituximab (anti-CD20) treatment for non-Hodgkin’s lymphoma has been shown to correlate with a polymorphism in FCγRIII, which is expressed by NK cells, showing that ADCC by NK cells might improve the effectiveness of some antibody-based cancer treatment regimens [141].

CD56bright CD16+ NK cells are the first to populate the bone marrow after bone-marrow transplantation and might shape the donor–transplant immune interaction [142]. The initial observation that relapse rates for leukemia after bone marrow transplantation were higher if the donor was an identical twin suggested that alloreactive NK cells promote a graft-versus-leukemia (GvL) effect [143]. A study assessed 1087 patients with acute myelogenous leukemia who underwent hematopoietic stem cell transplantation after reduced intensity conditioning [144]. The patients with donors with predominately activating KIR, rather than inhibitory KIRs, had a significantly reduced risk of relapse [144]. NK cells also reduce the rate of GvHD in allogeneic bone marrow transplants by killing host APCs and alloreactive T cells [145].

Overall, NK cells are promising tools for cancer immunotherapy and there are several NK-cell based therapies in clinical development, as has recently been extensively reviewed [146].

### 4.3. NK Cells in Pediatric Bone Sarcomas

In OS, when NK cells isolated from healthy and newly diagnosed patients with OS were stimulated with IL-15, they could lyse the OS cell lines effectively, including chemoresistant cell lines [147]. Furthermore, when developing a model for metastatic OS in nude mice, NK depletion increased the lung metastases dramatically, alluding to the importance of NK immune surveillance in metastasis prevention [148]. In another study, when NK cells isolated from healthy donors were first treated with IL-2 and then mixed with OS cell lines with KIR receptor/ligand mismatch, they lysed OS cells more effectively [149]. This outcome shows that making these inhibitory receptors unable to bind to a ligand substantially reduces the inhibition on NK cells [149]. In in vitro settings, it has been shown that human NK cells cocultured by feeder cells that express IL-15 (K562-mb15-41BBL) could effectively kill OS cell lines [10]. The same study also demonstrated the effective killing of EWS targets by activated NK cells. Therefore, in addition to OS, EWS cells are also susceptible to NK-mediated killing.

Cancer stem cells (CSC) are considered as the seed population of tumors that give rise to and maintain the tumor while being resistant to anti-proliferative therapies. Therefore, targeting CSCs could address the tumor problem and enhance the likelihood of long-term response to therapy. Allogeneic NK cells, when co-cultured with EBV-SMI-LCL feeder cells and treated with recombinant human IL-2, selectively killed the CSC population in A673, a soft tissue EWS cell line, in a fresh tumor sample and in a xenograft mouse model [150]. The increased CSC susceptibility to NK cells has been attributed to an increased expression of MICA/B (NKG2D ligands) and death receptors such as Fas [150]. Another study has shown that NK cells cultured with irradiated feeder cells that express IL-21 (K562-mb-IL-21) significantly decreased the lung metastasis in a xenograft mice model created with the metastatic EWS cell line, TC106 [151]. These activated NK cells did not affect the primary tumor growth, which is in contrast to other data in the literature. This difference could be due to the different phenotype and effector activities of the NK cells generated by the differing ex vivo activation protocols. These results cumulatively demonstrate that NK cells can target two key points responsible for relapse and mortality in EWS: the CSC population and metastasis. In a case report of a child with relapsed/refractory osseous EWS, when the FDA approved NK cell line, NK92 was administered intratumorally into the mandibular metastasis, the treatment was well tolerated and an apparent regression in the facial mass was achieved when this therapy was combined with vincristine, topotecan and cyclophosphamide chemotherapy [152]. These results underscore the power of combination therapies that involve NK cell immunotherapy and conventional therapy to effectively target these refractory tumors. Interestingly, a study showed that higher infiltration of NK cells in EWS could be a poor prognostic factor, which underscores the complexity of the tumor–NK cell interaction and the need to determine the phenotype and the effect of NK cells in various stages of the disease [153].

### 4.4. NK Cell Therapy in Pediatric Bone Sarcomas

Given that pediatric bone malignancies are ‘cold’ tumors that do not elicit potent T-cell responses and have thus far been unresponsive to ICI, NK cell therapy is a potential strategy for immunotherapy in these cancers. NK cells have several advantages over T cells as anti-tumor effectors. NK cells do not have MHC restriction, thereby making it easier to design “off-the-shelf” therapies that do not require the use of autologous cells. The risk of GvHD limits the use of allogeneic T cells, but given that NK cells do not generally pose such a risk, allogeneic cells could be used, which is especially important in pediatric patients with cancer, where invasive procedures should be minimized. NK cells also have multiple effector functions, such as cytolytic activity, ADCC and cytokine release. Given the susceptibility of these cancer cells to NK cells, developing NK cell therapies is a viable alternative to current treatment approaches. There are several completed and ongoing trials testing various adoptive NK cell therapy modalities in EWS and OS (Table 2).

**Figure 4:**
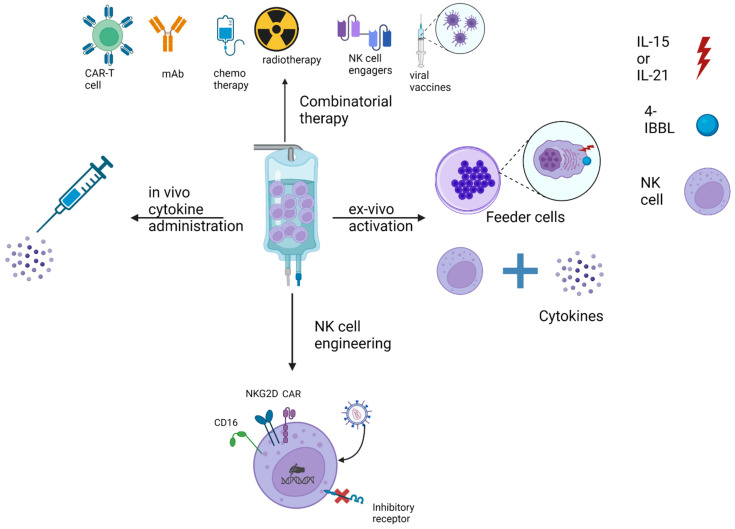
Approaches for an effective adoptive NK cell therapy in pediatric bone sarcomas. NK cells can be activated and expanded ex vivo by cytokines or membrane-bound cytokines presented on the feeder cells. Ex vivo activation by a cocktail of cytokines can also be employed to induce memory-like NK cells. The in vivo persistence and activation of NK cells can also be promoted by cytokine coadministration. NK cells are also engineered to express either higher quantities of activating receptor or their higher affinity forms, and to knock out inhibitory molecules/receptors. Inhibition is shown by the red cross in the figure. CAR-NK cells that are specific to tumor-associated antigens on sarcoma cells are also generated. The multieffector function of NK cells also make them suitable for combinatorial therapies because the cytokine released by these cells can stimulate other cells (such as CAR T cells), ADCC can increase monoclonal antibody efficiency, stress signals generated by chemotherapy and radiotherapy can enhance NK-cell recognition, and NK cells can effectively lyse tumor cells infected by viral vaccines.

The approaches employed in NK-cell-based ACTs are based on increasing NK-cell expansion, in vivo cytotoxic capacity, in vivo persistence, tumor infiltration and preventing their suppression and exhaustion by the inhibitory receptors and the TME (Figure 4).

For adoptive cell therapy, NK cells can be derived from numerous sources such as peripheral blood, hematopoietic stem cells, induced pluripotent stem cells (iPSC) and umbilical cord, each source having its own advantages and disadvantages.

#### 4.4.1. NK Cell Sources

There are diverse sources for NK cell ACT. Conventionally, the most widely used source has been autologous NK cells. Although this source has several advantages such as a lower risk of GvHD, it can fail to generate a potent response against cancer cells. New external sources for NK cell infusion have therefore been sought.

*Allogeneic peripheral blood NK cells*. NK cells account for on average,10% of all peripheral blood lymphocytes and can be obtained from healthy donors by leukapheresis [154]. Allogeneic NK cell adoptive transfer constitutes an accessible therapy and can provide good tumoral response, as Ruggeri et al., discovered in 1999 and 2002 when their patients with leukemia did not experience relapse and graft rejection [155]. Pérez-Martinez et al., subsequently studied this allogeneic therapy in solid tumors, showing encouraging results with all of their patients presenting some type of clinical response (complete response in 3 patients, partial response in 2 patients and stable disease in 1 patient after 9 months of follow-up) [156]. The disadvantages of this source is the risk of alloreactivity and multiple invasive procedures that might be required [157]. Due to the limited amount of NK cells in peripheral blood, several leukapheresis procedures and, in some instances, a central intravenous line might be required to obtain the necessary volume of blood, increasing the risk of complications for the donor [158].

*Umbilical cord blood (UCB) NK cells*. NK cells in UCB are more numerous than in peripheral blood given that, in the former, NK cells constitute 30% of all lymphocytes. UCB NK cells also have the advantage of greater accessibility through cord blood banks, which facilitates the selection of patients with certain HLA profiles and specific receptors. Spanholtz et al., employed cryopreserved UCB as a source of hematopoietic stem cells to produce NK cells derived from CD34+ cells in the biobanks [159]. It was also possible to expand these cells and produce an optimized and large production of highly functional NK cells for use in clinical trials [159]. It should be noted, however, that these cells have a more immature phenotype with a lower expression of adhesion molecules and activating receptors such as CD16, which translates into lower cytotoxic activity against the K562 leukemia cell line, despite producing similar amounts of IFN-γ and TNF-α as peripheral blood-derived NK cells [160].

*NK cell lines*. NK clonal cell lines are an alternative source of allogeneic NK cells. The NK-92 cell line, one of the most tested clonal lines, has demonstrated safety in various clinical trials and is FDA-approved for use. This line is an IL-2-dependent cell line and has the significant advantage of easy and reproducible expansion from a good manufacturing practice (GMP)-cryopreserved cell bank, with doubling times between 24 and 36 h to generate potent grade NK-92 effectors. However, its limitations include genetic instability requiring radiation, limiting their persistence to 48 h [161].

*Induced pluripotent stem cells*. These new sources are being studied and have emerged over time due to the drawbacks with other classical sources, such as donor variability and the leukocyte heterogeneity in their blood. The cost, time and difficulty involved in the cell extraction processes and the fact that conventional approaches involve intervention processes in a healthy donor are problematic [8]. Given these limitations, numerous research groups are developing this new type of therapy to make it possible to obtain virtually unlimited sources of homogeneous NK cells that are more susceptible to genetic manipulation regardless of the HLA haplotype [159]. These cells are known as iPSCs, which are generated by the initial reprogramming of adult cells towards pluripotency to allow their differentiation into NK cells [162]. In recent decades, several authors have studied this method of obtaining NK cells, observing greater cytotoxic activity than NK cells derived from UCB, while being just as effective as NK cells from peripheral blood in terms of increasing median survival for mice with different types of cancer [163,164].

#### 4.4.2. Promoting the Activation and Persistence of NK Cells

Similar to the ex vivo activation/priming of T cells for adoptive T-cell therapy, it has become apparent that NK cells fare better when activated ex vivo for an effective anti-tumor response. NK cell activation can be achieved by adding the cytokines IL-15 and/or IL-2 to the cultures. Membrane-bound interleukins expressed on the irradiated feeder cells such as K562mbIL15-41BBL that express costimulatory 4-1BB ligands and membrane-bound IL-15 are also being employed to generate activated and expanded NK cells. The clinical trial led by our group, which involves patients with refractory sarcoma (aged 0–30 years), also activated and expanded NK cells by K562mbIL15-41BBL since our preclinical studies in rhabdomyosarcoma have shown that tumor implantation was inhibited in vivo by these activated and expanded NK cells [82]. The same cell line, K562, is also being engineered to generate membrane-bound IL-21, K562mbIL21-41BBL, which is also being used as a feeder cell in NK-cell activation and expansion [165]. However, the use of feeder cells poses certain challenges, especially for compliance with GMP and safety. Although feeder cells are irradiated and theoretically cannot proliferate, given that they are cancerous cells, contamination of the final product by them (or their products’) might pose a safety risk, and extra steps are needed to ensure the absence of these cells in the final released product.

The short in vivo persistence of administered NK cells is one of the major impediments for the success of these therapies. Preconditioning by lymphoablative regimens is employed to provide a competitive advantage to the adoptively transferred cells to survive and increase in number. Given that these regimens put an extra strain on patients who have already been heavily treated, these harsh regimens need to be optimized through comparative studies to reduce the intensity of the conditioning to thereby increase the range of patients who could benefit from NK-cell-based ACTs.

To promote the in vivo persistence of these cells, IL-2 or IL-15 has been co-administered with NK ACT. An IL-15 superagonist complex (Alt 803) has been shown to promote NK and T-cell expansion without inducing Tregs, especially when administered subcutaneously in patients with relapsed leukemia and lymphoma [166]. A recently completed clinical trial tested the safety of infusing universal NK cells without HLA matching in combination with ALT-803 infusion in tumors including EWS (soft-tissue subtypes) and rhabdomyosarcoma (NCT02890758). We are currently running a sarcoma clinical trial using the infusion of haploidentical activated and expanded NK cells combined with the infusion of IL-2 to aid the in vivo persistence of the adoptively transferred cells. Another clinical trial had been testing a humanized anti-GD2 antibody linked to IL-2 (Hu14.18-IL2) in relapsed or refractory neuroblastoma and OS, administered along with NK cells activated and expanded ex vivo by K562mbIL15-41BBL, but the study was recently withdrawn because of “limited resources due to COVID-19” (NCT03209869). Nonetheless, this fusion protein has been found to be safe with reversible toxicities in children with neuroblastoma or GD-2+ tumors [167].

When nicotinamide (NAM) was added to the ex vivo NK-cell expansion cultures together with interleukins, it was shown that these cells persisted in NSG (NOD scid gamma) mice longer and proliferated more with less susceptibility to immune evasion [168]. Based on these results, a phase I clinical trial was conducted in patients with non-Hodgkin’s lymphoma and multiple myeloma where these cells were shown to persist and proliferate (NCT03019666). In the study, NK cells from allogeneic healthy donors were cultured ex vivo with nicotinamide and IL-15 and infused together with low-dose IL-2, after lymphodepleting therapy. To use the ADCC function of these cells, mAbs were also administered. The results showed that this approach was well tolerated with no neurotoxicity or cytokine release syndrome. There was a 73.3% clinical response in the patients with non-Hodgkin’s lymphoma, who had already been heavily treated [169]. All in all, this shows that NK cell potency can be maximized by optimizing ex vivo and in vivo NK-cell-promoting conditions.

One of the more interesting and unexpected approaches that has emerged for increasing the in vivo persistence and cytotoxicity of NK cells is the generating of memory-like NK cells. Although NK cells have classically been considered members of the innate immune system, increasing evidence has shown that NK cells can acquire “memory-like” properties after hapten exposure, virus infection or preactivation by an interleukin cocktail [170]. The memory-like functional features of NK cells are defined by elevated functional activity and the ability to generate specific recall responses.

Cytokine-induced memory-like (CIML) NK cells can be generated by a brief preactivation of the cells by a combination of cytokines [171]. CIML NK cells have been characterized by enhanced IFN-γ production, higher killing potency and proliferation [171]. This approach is marked by the simplicity of its protocol for generating these effective cells, requiring only a 16-h long incubation with a mix of three cytokines (IL-12, IL-15, and IL-18) followed by a 6-day culturing of the cells with a low level of IL-15. There is therefore no need for feeder cells, which facilitates its translation to clinical practice under GMP conditions. It has been shown that this brief cytokine exposure leads to epigenetic changes at the IFN-γ locus, which results in increased IFN-γ production and longer in vivo persistence [172]. Our laboratory is currently running in vitro experiments to test CIML NK cells against EWS and OS cells, with encouraging results. However, the primary inhibitory NK-cell receptor, CD94-NKG2A, is also upregulated in these cells, and effective anti-tumor activity might require the blocking of this receptor, along with the administration of CIML NK [173]. A phase II study that combined CIML NK infusion with hematopoietic cell transplantation from the same HLA-haploidentical donor and included the infusion of a IL-15 superagonist in patients with relapsed/refractory acute myeloid leukemia showed that CIML NK cells can persist in vivo for more than 2 months (NCT02782546) [174]. Despite the lack of any current clinical trials using CIML NK cells in pediatric sarcomas, our preliminary studies show that CIML NK-based therapies hold promise for pediatric sarcomas, especially EWS.

In addition to CIML, natural adaptive-like NK cells have been shown to be present in the peripheral blood of human cytomegalovirus (CMV)-positive individuals [175]. These cells are characterized by the expression of the stimulatory receptor NKG2C, which recognizes CMV-specific peptides presented on HLA-E and displays a more potent cytotoxic response [176]. When NK cells from CMV+ haploidentical donors were cultured along with IL-15 and the GSK3b inhibitor (an inhibitor that augments the cytolytic and ADCC activity of NK cells), and then CD3− CD19− CD57+ NKG2C+ FATE-NK100 cells were administered intraperitoneally to patients with refractory ovarian cancer, these cells persisted in the ascites for more than 20 days (NCT03213964) [177]. These results cumulatively underscore the vast potential of NK-cell potency that could be unlocked and augmented by various means and emphasizes that every step of NK-cell preparation (from culture conditions to donor selection) has a substantial effect on the characteristics of the infused NK cells and should be closely monitored and refined.

#### 4.4.3. NK Cell Engineering

Advances in genetic engineering and biotechnological methods have enabled the generation of “designer” immune cells with desired specificities. As has been previously mentioned, CAR-T cells produced by transducing a chimera of an antibody subdomain (single-chain variable fragment) and TCR activation (and costimulatory) motifs have shown substantial clinical success in hematological cancers, such as multiple myeloma and B-cell malignancies.

The success of CAR-T therapy has not yet been translated to solid tumors due to a multitude of reasons such as poor tumor infiltration and immunosuppressive TME. GD-2-specific CAR-T cells, as well as CAR-T cells specific to other sarcoma markers, CD133, HER2, Muc1 and CD117 are being tested in clinical trials (NCT03356782, NCT03356782, NCT04433221). Therapeutic approaches in sarcoma based on modified T cells have recently been reviewed [178]. However, the HLA restriction of T-cell-based therapies to prevent GvHD, limits the streamlining of the manufacturing processes and limit the therapy mainly to autologous sources. Furthermore, long manufacturing times impede the use of this approach in rapidly progressing disease settings.

CAR-NK cells circumvent these problems because there is no need for HLA matching, and therefore, off-the-shelf universal CAR-NK cells can be produced. A wide variety of intrinsic activating receptors on NK cells can also enhance the anti-tumoral activity of the construct. The fact that NK cells are short-lived can limit the toxicity associated with these treatments but inevitably this can have repercussions on their efficacy.

When NK cells isolated from patients with multiple myeloma and from healthy donors were activated and expanded ex vivo and transduced with NKG2D CAR by a lentiviral construct, multiple myeloma growth was abrogated in NSG mice, where CD45RA- CAR-T cells failed to show a substantial effect [179]. This CAR construct provides an enhanced affinity for NKG2D ligands, such as MICA/B, expressed on the solid tumors. A clinical pilot study to test the feasibility and safety of using NKG2D CAR-NK cells in metastatic solid tumors is currently recruiting patients (NCT03415100). In an interesting study that combined CAR-NK and CAR-T cells, NKG2D-specific CAR-NK cells were generated to eliminate the NKG2DL-expressing suppressive MDSCs in TME, to lift the immunosuppression on the GD-2-CAR-T cells, and this approach allowed CAR-T cells gain functionality against the tumor cells [180]. CAR-NK cells for another main activating NK cell receptor, DNAM-1, have also shown enhanced cytokine release and degranulation against the erythroleukemic cell line [181].

CAR-NK cells specific to tumor-expressed antigens have also been produced. GD-2-specific CAR-NK cells showed enhanced in vitro response to EWS cells but failed to mount an effective response to EWS xenografts due to upregulation of immunosuppressive HLA-G [182]. Given that inhibitory receptors are an integral part of NK cell biology, it is possible that NK-cell-activating approaches should be coupled with inhibitory receptor blocking to produce the net positive stimulus for NK-cell activation.

A clinical trial was recently launched to test the safety and optimal dose for cord-blood-derived CD70-targeting CAR-NK cells that are also transduced with IL-15 in three advanced solid tumors, including OS (NCT05703854). Other CAR-NK-cell targets that could have a potential use in pediatric bone sarcomas include HER-2 and CD133 (NCT04319757). There is also an ongoing trial testing CAR-NK cells targeting ROBO1, the receptor for Slit2, that has shown to be important in EWS growth and associated with poorer survival in OS, in adults with ROBO1-expressing solid tumors (NCT03940820) [183].

The targeting of metastatic disease is one of the viable strategies for increasing survival. The MUC-1 protein has been associated with sarcoma metastasis, and there is a current clinical trial using CAR-NK cells targeting MUC-1 in adult solid tumors (NCT02839954) [184]. CAR-NK cells targeting IGF1R can also be a potential immunotherapy approach in pediatric bone sarcomas.

NK cells are hard to transfect/transduce, with low transgene expression efficiency and reduced viability upon the procedure. Most studies have employed the NK92 cell line for viral transduction to produce engineered NK cells. Gene transfer into NK cells is usually performed through lentiviral or retroviral vectors. Viral vectors have higher safety risks such as potential immune response against the viral particles. Transient gene expression via electroporation is also being employed, as in the aforementioned NKG2D-CAR-NK cell clinical study, where NKG2D mRNA is electroporated in autologous and allogeneic NK cells (NCT03415100). Transposon systems such as PiggyBac transposons that transpose the gene of interest to the NK cell genome are also being studied.

The clustered regularly interspaced short palindromic repeats (CRISPR) CRISPR/Cas9 system is the newest addition to genome editing technologies and has found widespread use in biomedicine due to its high efficiency, low production cost, suitability for mass production, flexibility, and ease of use [185]. This new and powerful genome editing technology can be used to knock in genes such as high-affinity CD16 for enhanced ADCC into NK cells and to knock out inhibitory receptors such as NKG2A and TIGIT in NK cells to increase their anti-tumoral capacity [186,187]. Another use could be the prevention of fratricide in CAR-NK cells. For example, given that CD70 is also upregulated in activated NK cells, CD70 expression in CD70 CAR-NK cells has been successfully knocked out by CRISPR/Cas9 technology to prevent fratricide by the CAR cells [188].

#### 4.4.4. Combination Therapies

Another important approach for enhancing the anti-tumoral efficiency of adoptively transferred NK cells is combination therapy. NK-cell ACT can be combined with conventional sarcoma therapy, especially as mentioned in the previous section with therapies that induce stress signals (the ligands of activating NKG2D receptor and DNAM-1). Accordingly, the treatment of multiple myeloma cells with doxorubicin increased the ligand expression of these receptors and led to an enhanced NK-cell degranulation [189]. Muramyl tripeptide phosphatidyl ethanolamine (mifamurtide) is a synthetic lipophilic molecule derived from the muramyl dipeptide found in bacteria. Similar to its parent natural molecule, this molecule can stimulate macrophage activation and is approved for use in nonmetastatic OS [190]. Mifamurtide stimulates macrophages to secrete cytokines and establishes an immune-primed environment. The combination of mifamurtide with NK-cell therapy could promote synergistic effects by engaging multiple immune effectors and promoting the release of proinflammatory cytokines, similar to a natural immune response.

Given the ADCC carried out by NK cells, one of the obvious types of combinational therapy is with mAbs. In particular, combining ICI with NK-cell therapy could synergistically boost the efficiency of both therapies. One of the challenges in immune-based combination therapies is the risk of immune-related adverse effects. However, the combination of autologous NK cells and mAbs against PD-L1 has been shown to be well-tolerated in patients with advanced sarcoma who have been heavily pretreated (NCT03941262). CIML NK cells have a higher ADCC capacity, and several studies have employed this treatment by combining CIML NK ACT with antibodies. A clinical study is currently evaluating the safety and efficacy of CIML NK cells combined with IL-15 superagonist and ipilimumab in advanced head and neck cancer (NCT04290546). The safety profile of this trial is of significant interest because the immune response is being stimulated on three fronts. Other mAbs that could potentially be combined with NK cell ACT include antibodies targeting the inhibitory NK receptors such as anti-NKG2A (e.g., monalizumab) and anti-KIR2D (e.g., lirilumab) and antibodies targeting TAAs, such as anti-IGF1-R (e.g., cixutumumab).

Similar to bispecific T-cell engagers (BiTe), bispecific antibodies that bind to a NK surface molecule and a tumor antigen (BiKe) are also being developed and studied. AFM13 is a NK-cell engager with CD16 and CD30 specificity; an in vivo xenograft lymphoma model demonstrated that when cord-blood-derived NK cells (preactivated into a memory-like phenotype and expanded) was loaded with AFM13, tumor growth was controlled, and survival was increased [191]. AFM24, which targets NK cells and EGFR, has been combined with NK cells for EGFR-expressing tumors and is undergoing testing in a clinical trial after its safety was confirmed in cynomolgus monkeys and its effectiveness was shown in other preclinical studies (NCT05099549) [192].

In addition to mAbs, NK cells can be combined with other immunotherapies such as DC and protein vaccines. DC vaccines from autologous or allogeneic cells can be generated by pulsing these professional antigen-presenting cells with tumor lysates (NCT01803152), individual TAAs, recombinant TAAs or by transfecting TAA-coding mRNAs. DC vaccines work mainly by activating effector T cells, and the combining this T-cell activation with NK-cell activation can potentiate the tumor lytic activity. There is an ongoing trial that is combining sarcoma vaccines with low-dose chemotherapy and CAR-T-cell infusion in patients with EWS and OS (NCT04433221). Similar studies are likely to be designed that combine DC vaccines with NK or CAR-NK cells for sarcoma.

In addition to DC vaccines, direct infusion of TAAs is another approach, albeit a weaker one. However, Badrinath et al., recently showed that when mice were injected with nanoparticles formed from the MICA/B α3 domain, T and NK cells were potently activated, and the mice with melanoma and breast cancer were protected from metastatic disease upon the removal of the primary tumors [193].

Oncolytic viral therapy is one of the most advanced innovative approaches in pediatric sarcomas. Oncolytic viruses are viruses that preferentially proliferate and lyse tumor cells while sparing the healthy cells. The subsequent tumor lysis also stimulates immune responses. Many different types of naturally occurring or engineered viruses are being tested in sarcomas [194]. One particularly interesting engineered viruses that could be combined with NK cell therapy, is oncolytic human herpes simplex virus type 1, which also expresses IL-12 and anti-PD-1 antibody and is currently being tested in adults with advanced solid tumors, including sarcomas (NCT05602792).

Overall, NK cells provide a versatile therapeutic platform in pediatric bone sarcomas with the availability of multiple sources of cells, the potential to use allogeneic cells, and the ability to apply a variety of engineering approaches and combination treatments. NK cells thus provide a promising option for improving outcomes in the treatment of refractory pediatric bone sarcomas.

## 5. Conclusions and Future Perspectives

Pediatric bone sarcomas are important childhood/adolescent tumors that are associated with high mortality, and their survival rates have plateaued despite an unprecedented rate of advancements in oncology. The conventional multimodal therapies employed for these types of cancer have debilitating effects on the quality of life of the patients. Therefore, there is a pressing need for safe and effective therapies that do not just kill the cancer cells but cure the patients, allowing them to thrive in life without treatment-associated long-term effects.

Although immunotherapy has revolutionized the treatment for certain types of cancer, its potential has yet to be realized in pediatric bone sarcomas. Sarcoma cells are particularly susceptible to NK-mediated killing. NK cells, as potent anti-tumor effectors without HLA restriction or need for prior sensitization, are an important candidate for tapping into the potential of immunotherapy in these cancers. The discovery of the memory-like properties of these innate effectors demonstrates that there is still much to be learned about these cells. Given the variety of potential sources and the safety of allogeneic NK-cell transfusions, it is feasible to have universal and easily accessible NK-cell therapies. However, ex vivo culturing, and cryopreservation techniques still need to be optimized and validated to ensure a robust NK cell supply. In allogeneic transfusions, delineating the donor properties both in terms of NK-cell phenotype and genetic match/mismatch with the recipient would boost the therapy effectiveness.

The engineering of NK cells to increase tumor infiltration and cytotoxic activity and decrease the effect of TME suppression is being used to ameliorate the shortcomings of NK-cell ACT. The refining of NK-cell engineering approaches to optimize safety, cost and scalability, especially in a GMP-compliant manner, would help in translating these approaches to clinical practice.

To tailor NK-cell therapies for pediatric bone sarcomas, we need to combine the latest technologies such as artificial intelligence, genome engineering and organ-on-chip platforms, with knowledge on tumor and NK-cell biology. The TAAs that are differentially expressed in sarcoma cells and are derived from proteins that are vital to the tumor cells to prevent immune escape need to be investigated by employing large databases and bioinformatics tools. An increased understanding of NK-cell activation, trafficking and phenotypes is also needed to refine the therapeutic protocols in terms of the choice, duration and quantity of cytokines used. The sequencing of tumors from sarcoma patients and use of tools such as artificial intelligence to devise the best combination therapy could lead to effective personalized approaches in these heterogeneous tumors. Given the low number of patients per center, multinational and multicenter consortiums need to be formed to obtain a comprehensive library of tumor samples.

Overall, we have only scratched the surface of the potential of NK cells in pediatric bone sarcomas, and this could be the dawn of an exciting NK cell era for pediatric bone sarcoma therapies.

## Figures and Tables

**Table 1:** Differential Characteristics of Osteosarcoma and Ewing Sarcoma.

	Osteosarcoma	Ewing Sarcoma
**Age at onset**	Second decade of life	1–10 years
		Second decade of life
**Sex (M:F)**	1.6:1	1.5:1
**Predisposing factors and associated syndromes**	Retinoblastoma, Paget disease, Li-Fraumeni syndrome, Rothmund-Thomson syndrome and Bloom syndrome	None
**Location**	80% extremities, 20% axial skeleton	50–60% large bones, 45% axial skeleton, more soft tissue involvement
**Bone involvement**	Metaphysis of long bones (femur, tibia and humerus)	Diaphysis of large bones Flat bones
**Symptoms**	Swelling, masses and subsequent pain	Pain, swelling, masses, fever (20%) and systemic symptoms are more frequent
**Metastasis**	Lung, bones	Lung, bones, bone marrow
**Treatment**	Phase I: Neoadjuvant chemotherapy	Phase I: Neoadjuvant chemotherapy
	Phase II: Surgery	Phase II: Surgery and/or radiotherapy
	Phase III: Adjuvant chemotherapy	Phase III: Adjuvant chemotherapy and/or radiotherapy and/or autologous transplantation of hematopoietic progenitors
**Prognostic factors**	Tumor volume, axial location, metastasis at diagnosis, alkaline phosphatase elevation, poor response to neoadjuvant chemotherapy (<90% of necrosis)	Metastasis at diagnosis (more important), axial location, tumor volume > 200 mL, maximal diameter > 8 cm, older age, male sex, LDH elevation, gene expression profile (p53, Ki67 overexpresion, 16q loss), poor response to neoadjuvant chemotherapy (<100% of necrosis)
**Survival**	Undisseminated: 60–74%, Disseminated: 30%	Undisseminated: 60–75%, Disseminated: 20–30%

**Table 2:** Completed and ongoing clinical trials with adoptive NK cell therapy in EWS (osseous) and OS (Abbreviations: EWS, Ewing sarcoma; HLA, human leukocyte antigen; IL, interleukin; NK, natural killer; OS, osteosarcoma; TGF, transforming growth factor).

Identifier	Cancer Type	Intervention	Phase	Status	Pediatric/Adolescent Population Included?
NCT05703854	Advanced OS	Cord-blood-derived CD70-specific CAR NK cells transduced with IL-15 with lymphodepleting chemotherapy	I/II	Not yet recruiting	No
NCT05634369	Relapsed/Refractory OS and EWS	Universal donor NK cell infusions that have been ex vivo expanded and cultured with TGF-β (TGF-β imprinted), with Gemcitabine and Docetaxel	I/II	Recruiting	Yes
NCT03420963	Recurrent and Refractory OS and EWS	Infusion of expanded cord-blood-derived allogeneic NK cells, cyclophosphamide and etoposide	I	Recruiting	Yes
EUDRACT 2016-003578-42	Refractory OS and EWS	Haploidentical ex vivo activated and expanded NK cells and IL-2 administration after lymphoablative chemotherapy, and radiotherapy	I/II	Recruiting	Yes
NCT02100891	OS and EWS	Haploidentical hematopoietic cell transplantation followed by adoptive transfer of donor NK cells on posttransplant day 7	II	Active, not recruiting	Yes
NCT01875601	Refractory sarcoma	Autologous NK cell infusion with or without recombinant human IL-15 administration	I	Completed	Yes
NCT02890758	EWS	Ex vivo expanded HLA mismatched NK cell therapy with or without IL-15 superagonist (ALT-803)	I	Completed	No
NCT01386619	EWS and OS	NK cell-enriched donor lymphocyte infusion following HLA-haploidentical hemopoietic stem cell transplantation	I/II	Completed	Yes

## Data Availability

Not applicable.
